# Supplementary material for: The Economic Implications of Phasing Out Pig Tail Docking: A Pilot Study in Italy
Source: Animals (Basel). 2025 Apr 29;15(9):1250. doi: 10.3390/ani15091250 (PMC12070853; doi:10.3390/ani15091250)
Supplement: Supplementary file 1 [file animals-15-01250-s001.zip › animals-3576044-supplementary.pdf]

animals

MDPI

Supplementary materials

The economic implications of phasing out pig tail docking: a pilot study in Italy

Francesca Menegon [1], Annalisa Scollo [2], Samuele Trestini [3,*], Rachele Urbani [1], Giuseppe Ru [4] and Guido Di Martino [1]

[1] Istituto Zooprofilattico Sperimentale delle Venezie, Viale dell'Università 10, 35020 Legnaro, Italy
[2] Department of Veterinary Sciences, University of Torino, Grugliasco, 10095 Torino, Italy
[3] Department of Land, Environment, Agriculture and Forestry (TESAF), University of Padova, 35020 Legnaro, Padova, Italy
[4] Epidemiology Unit, Istituto Zooprofilattico Sperimentale del Piemonte, Liguria e Valle D'Aosta, Via Bologna 148, Torino, Italy

* Correspondence: samuele.trestini@unipd.it

Table S1. Time flow of the study. Number of batches observed per year, step and growing phase are shown.

Growing phase	2015	2016	2017	2018	2019	2020	2021
Weaning							
Step 1					10	6	
Step 2						4	4
Step 3							3
Fattening							
Step 1	2	15	24	12	8	4	
Step 2				3	2	2	
Step 3				1	2	4	4

Table S2. Overview of the risk factor mitigation strategies - other than environmental enrichment - implemented during steps 2 and 3 of the study (tail step: 2 = 10-50% undocked pigs; 3 = 100% undocked pigs) across the pig farms involved in the study (identified by letters a-v) to reduce the incidence of tail biting. Each column represents a specific risk factor identified by EFSA [3], while each row corresponds to a farm. A marked cell indicates that the farm has implemented measures to reduce that specific risk factor.

Farm	Stocking density	Microclimate	Ventilation	Trough space	Balanced diet	Feeding timeliness	Drinkers/pigs ratio	Disease prophylaxis	Mixing pigs	Behavioural monitoring
Weaning										
a	X	X	X		X	X	X	X		X
b	X			X	X			X	X	
c	X	X	X		X		X	X		
d	X				X			X	X	
Fattening										
e										X
f	X									X

g			X					X		X
h	-	-	-	-	-	-	-	-	-	-
i	X					X				X
j	X							X		
k	X									
l	X									
m	-	-	-	-	-	-	-	-	-	-
n	X					X				
o	X			X						
p	-	-	-	-	-	-	-	-	-	-
q					X			X		
r	-	-	-	-	-	-	-	-	-	-
s	X	X				X				X
t	-	-	-	-	-	-	-	-	-	-
u	-	-	-	-	-	-	-	-	-	-
v	-	-	-	-	-	-	-	-	-	-

Table S3. Results of linear regression between mortality (independent variable, mean %) and costs (€) per kg of produced meat (dependent variable). The number of batches examined in the weaning phase was 36, and 84 in the fattening. The table includes the constant coefficient, the mortality coefficient, the respective standard errors (St. e.) and significance (*p*-value), and the coefficient of determination (R^2).

	**Constant**			**Mortality**			**R^2**
	coefficient	St. e.	P-value	coefficient	St. e.	P-value	
Weaning							
Cost of feed*	0.98	0.03	<0.001	0.03	0.00	<0.001	0.84
Cost of piglets purchasing*	1.51	0.08	<0.001	0.11	0.01	<0.001	0.90
Cost of drugs*	0.04	0.01	<0.001	0.01	0.00	<0.001	0.67
Contractor fees*	0.14	0.02	<0.001	0.02	0.00	<0.001	0.77
Total cost (excluding VAT)*	2.68	0.10	<0.001	0.17	0.01	<0.001	0.92
Feed cost for 100 pigs	3030.69	93.84	<0.001	-6.33	7.82	0.424	0.02
Feed yield (%)	48.92	0.78	<0.001	-0.33	0.07	<0.001	0.43
Fattening							
Cost of feed*	0.70	0.01	<0.001	0.01	0.00	<0.001	0.23
Cost of pigs purchasing*	0.54	0.0	<0.001	0.01	0.00	<0.001	0.35
Cost of drugs*	0.01	0.00	<0.001	0.00	0.00	0.107	0.03
Contractor fees*	0.14	0.01	<0.001	0.00	0.00	<0.001	0.24
Total cost (excluding VAT)*	1.39	0.01	<0.001	0.03	0.00	<0.001	0.72
Feed cost for 100 pigs	11,315.85	422.71	<0.001	219.95	60.18	0.001	0.14
Feed yield (%)	33.03	0.59	<0.001	-0.47	0.09	<0.001	0.27

*€/kg of meat produced
